# Case Report: Five-year follow-up of leptomeningeal transthyretin amyloidosis (Ala45Thr) with CNS involvement under tolcapone therapy

**DOI:** 10.3389/fmed.2026.1853003

**Published:** 2026-07-22

**Authors:** Nataša Giedraitienė, Eglė Preikšaitienė, Aušra Klimašauskienė

**Affiliations:** 1 Faculty of Medicine, Clinic of Neurology and Neurosurgery, Institute of Clinical Medicine, Vilnius University, Vilnius, Lithuania; 2 Department of Human and Medical Genetics, Faculty of Medicine, Institute of Biomedical Sciences, Vilnius University, Vilnius, Lithuania

**Keywords:** CNS involvement, leptomeningeal, p.Ala45Thr pathogenic variant, tolcapone, transthyretin amyloidosis

## Abstract

**Background:**

Transthyretin (TTR) p.Ala45Thr variant is pathogenic, typically associated with cardiomyopathy and peripheral neuropathy. Leptomeningeal and central nervous system (CNS) involvement is rare, and longitudinal treatment data remain limited.

**Methods:**

We conducted a single-patient longitudinal observational study of a patient with genetically confirmed hereditary transthyretin amyloidosis (ATTRv) carrying the *TTR* p.Ala45Thr variant with CNS involvement. The patient was followed over 5 years using multimodal assessments, including neurological and cognitive testing, cerebrospinal fluid (CSF) analysis, magnetic resonance imaging (MRI), and electrophysiological studies.

**Results:**

A 48-year-old woman presented with progressive neurological symptoms beginning at age 37, including gait ataxia, dysarthria, and left hypoesthesia. Family history was notable for early-onset neurological decline in maternal relatives. Baseline MRI showed cerebellar micro-hemorrhages, leptomeningeal enhancement, and a cervical spinal cord lesion (C4–C7). CSF protein was markedly elevated, and genetic testing confirmed the TTR p.Ala45Thr variant. Cardiac and peripheral nerve evaluations were unremarkable. Tolcapone therapy was initiated and titrated to the maximal dose. Over 5 years, follow-up assessments demonstrated progressive neurological and cognitive decline, increased CSF protein levels, and enlargement of the spinal cord lesion, with mild progression of leptomeningeal and ependymal enhancement on MRI.

**Conclusion:**

This report highlights progressive CNS involvement in Ala45Thr-associated ATTR despite treatment with a CNS-penetrant TTR stabilizer. These findings suggest that disease progression may continue despite TTR stabilization in some patients and underscore the need for early intervention, systematic CNS monitoring, and further development of CNS-targeted therapies.

## 1 Introduction

Hereditary transthyretin amyloidosis (ATTRv) usually manifests as progressive peripheral neuropathy or restrictive cardiomyopathy, but may also involve the kidneys, gastrointestinal tract, eyes, and leptomeninges (1–3). Over 200 different *TTR* pathogenic variants are known (4). Many of these variants show distinct tissue tropisms and variable clinical severity, emphasizing the heterogeneity of ATTRv. While genotype–phenotype correlations summarized in the literature have suggested a potential association between certain TTR variants and leptomeningeal involvement, detailed clinical and longitudinal reports describing central nervous system (CNS) or leptomeningeal manifestations are scarce (5, 6). Treatment options for CNS involvement remain limited, as current pharmacological approaches—such as TTR tetramer stabilizers (including tolcapone and preclinical candidates such as PITB)—or liver transplantation do not halt production of mutant TTR within the CNS, and all available data on these therapies come from short-term pharmacodynamic or proof-of-concept studies, with long-term clinical outcomes remaining unknown (7–13). Structural and biophysical analyses reveal that tolcapone binds with high affinity and specificity to three unstable leptomeningeal *TTR* variants, stabilizing them and thereby preventing aggregation (9–11, 14–16). All available data on the treatment of leptomeningeal ATTR come from short-term pharmaco-dynamic or proof-of-concept studies (9–11, 16), but long-term clinical outcomes remain unknown.

Here, we describe a patient with the TTR (NM_000371.4) c.133G>A variant resulting in substitution of threonine for alanine p.Ala45Thr. The patient began tolcapone treatment in 2021. We present mechanistic insights from a 5-year longitudinal follow-up, including neurological, cognitive and functional testing, cerebrospinal fluid (CSF) analysis, cranial and cervical spine magnetic resonance imaging (MRI), and evoked potentials, highlighting disease progression and treatment response. This clinical report provides a novel perspective on the neurological manifestations of a rarely reported variant and the potential mechanistic impact of tolcapone therapy in ATTRv.

## 2 Methods

### 2.1 Study design and patient

This was a single-patient longitudinal observational study of a patient with genetically confirmed hereditary transthyretin amyloidosis (ATTRv) carrying the TTR c.133G>A (p.Ala45Thr) variant with CNS involvement. The patient was followed at Vilnius University Hospital Santaros Clinics over a 5-year period during routine clinical follow-up as part of multimodal disease monitoring.

### 2.2 Functional and cognitive testing

Clinical and cognitive assessments were performed by the same examiner at all study time points to ensure consistency. Functional evaluation included the 9-Hole Peg Test (9HPT), performed for both dominant and non-dominant hands, and the 25-Foot Walk Test (25FTW). Cognitive function was assessed using the Symbol Digit Modalities Test (SDMT, oral version), Brief Visuospatial Memory Test–Revised (BVMT-R), and California Verbal Learning Test–Second Edition (CVLT-II).

### 2.3 Neuroimaging

Magnetic resonance imaging (MRI) of the brain and cervical spine was performed using a Philips Achieva 3 T scanner (software release 5) according to a standardized clinical protocol. The protocol included T1-weighted 3D imaging, T2-weighted turbo spin echo (TSE), fluid-attenuated inversion recovery (FLAIR), diffusion-weighted imaging (DWI), susceptibility-weighted imaging (SWI/VEN BOLD), and post-contrast T1-weighted sequences. All images were evaluated by a neuro-radiologist using identical acquisition parameters throughout the follow-up period.

### 2.4 Cerebrospinal fluid analysis

CSF samples were obtained via lumbar puncture and analyzed using standard laboratory techniques. Parameters included total protein concentration, albumin, immunoglobulin G (IgG), IgG index, oligo-clonal bands, and cell count (cells/μL).

### 2.5 Electrophysiological studies

Nerve conduction studies (NCS), somatosensory evoked potentials (SSEPs), and electroencephalography (EEG) were performed using standard clinical neurophysiology protocols. SSEPs were recorded bilaterally following posterior tibial nerve stimulation and analyzed for central conduction time.

### 2.6 Systemic and ophthalmological evaluation

Cardiac evaluation included electrocardiography and echocardiography with strain analysis. Ophthalmological examination included visual acuity testing, fundus examination, optical coherence tomography (OCT), and retinal nerve fiber layer thickness assessment.

## 3 Results

The patient is a 48-year-old woman who first presented to our center at the age of 43. She had first experienced neurological symptoms at the age of 37. At her initial evaluation in our center, neurological examination revealed gait ataxia, dysarthria, left-sided hypoesthesia, bilateral pathological reflexes, urinary urgency, and marked nuchal rigidity. Two self-limited episodes of encephalopathy, occurring 2 years and 1 year prior to presentation, were documented in her medical records; both resolved completely without treatment.

Six years prior to presentation, a cervical spine MRI performed at an outside hospital showed no abnormalities. Four years ago, CSF analysis at the same institution revealed a protein concentration of 1.86 g/L and a mildly elevated cell count (22 cells/μL). Two years ago, a follow-up MRI at a local hospital demonstrated an intramedullary lesion at C4–C7. Treatment with antibiotics and high-dose corticosteroids did not result in any improvement.

**Figure 1:**
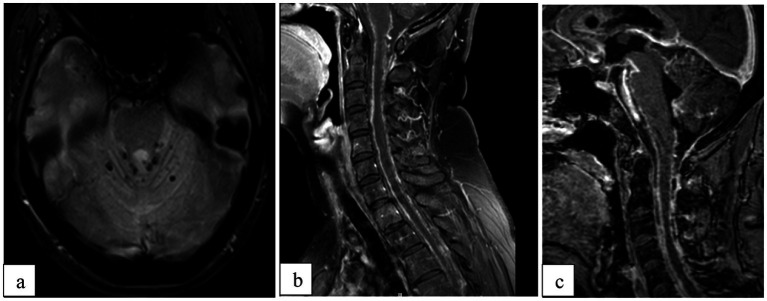
**(a)** Baseline T2-W GRE MRI at the level of the posterior fossa demonstrates multiple hypo-intense foci in the cerebellar hemispheres consistent with micro-bleeds. **(b)** Baseline T1-W sagittal MRI of the spinal cord with gadolinium contrast demonstrates diffuse leptomeningeal enhancement involving the cervical spinal cord and brain, with a hyper-intense lesion in the central cord at C4–C7 consistent with myelopathy. **(c)** Follow-up T1-W TFE sagittal MRI with gadolinium contrast after 5 years demonstrates slight increase of the cervical cord myelopathic lesion (now extending to C7), with persistent leptomeningeal enhancement from the foramen magnum to Th4. MRI, magnetic resonance imaging; T2-W GRE, T2-weighted gradient echo; T1 W, T1-weighted; TFE, turbo field echo; C4–C7, Th4, cervical (C4–C7) and thoracic (Th4) vertebral levels.

At the time of diagnosis, cranial MRI showed multiple small micro-hemorrhagic foci, more prominent in the cerebellum, with leptomeningeal contrast enhancement predominantly in the sub-tentorial region, consistent with leptomeningeal amyloidosis. Cervical spine MRI demonstrated a dorsal T2 hyper-intense area along the midline of the spinal cord from C4 to C7, with contrast enhancement. Diffuse meningeal thickening persisted, with possibly increased vascularity around the spinal cord, medulla, pons, and cerebellum (Figure 1). Lumbar puncture revealed a cerebrospinal fluid protein level of 6.49 g/L and mildly elevated cells (Table 1). Next generation sequencing analysis of genomic DNA was performed as described previously (17). Genes associated with adult-onset neurodegenerative disorders were analyzed. A missense variant NM_000371.3:c.133G>A, NP_000362.1:p.Ala45Thr in the TTR gene was identified.

**Table 1:** Baseline and longitudinal assessments of clinical, cognitive, laboratory, and radiological parameters.

Parameter	Baseline	Year +1	Year +2	Year +3	Year +4	Year +5
Clinical scores						
9HPT-D*	20.3	21.0	21.8	24.8	32.3	37.4
9HPT-N*	21.2	22.1	22.5	26.9	38.6	41.9
25FTW**	4.5	5.8	6.8	7.4	11.2	18.9
Cognitive assessment						
SDMT***	59	58	54	55	50	45
BVMT-R****	30	29	26	25	25	23
CVLT-II*****	62	55	50	50	48	44
CSF parameters						
CSF protein (g/L)	6.49	22.31	39.0	NA	NA	64.53
IgG (mg/L)	639.41	NA	3746.78	NA	NA	6155.81
Albumin (mg/L)	3574.9	NA	21905.5	NA	NA	34274.6
IgG index	0.70	0.69	0.71	NA	NA	0.75
OCB	Absent	Absent	Absent	NA	NA	Absent
Cells (cells/μL)	22	24	47	NA	NA	28
Electrophysiological, ophthalmologic, and cardiac evaluations						
NCS	Normal	Normal	Normal	NA	NA	Normal
SSEPs-R (ms)	42.6	42.2	41.9	NA	NA	41.2
SSEPs-L (ms)	43.8	44.1	50.5	NA	NA	61.5
EEG	Normal	Normal	Normal	NA	NA	Normal
Ophthalmological exam******	Normal	Normal	Normal	Normal	Normal	Normal
Cardiac assessment*******	Normal	Normal	Normal	Normal	Normal	Normal
MRI imaging						
Cranial MRI	Multiple small micro-hemorrhages, leptomeningeal enhancement	Stable, no new changes	Stable, no new changes	Increased leptomeningeal enhancement, slightly more intense ependymal enhancement	Stable, no new changes	Stable, no new changes
Spinal MRI	C4–Th1 myelopathic lesion, leptomeningeal enhancement	C4-C7 myelopathic lesion enlargement, marked leptomeningeal enhancement	C4-C7 myelopathic lesion with increased volume, leptomeningeal enhancement	C4-6 myelopathic lesion	Iki C3-Th1 myelopathic lesion, marked leptomeningeal enhancement	C4-7 myelopathic lesion (slightly longer), leptomeningeal enhancement FM–T4

9HPT-D, 9-Hole Peg Test (dominant hand); 9HPT-N, 9-Hole Peg Test (non-dominant hand); 25FTW, 25-Foot Walk; SDMT, Symbol Digit Modalities Test; BVMT-R, Brief Visuospatial Memory Test–Revised; CVLT-II, California Verbal Learning Test, Second Edition; CSF, cerebrospinal fluid; Ig, immunoglobulin; OCB, oligoclonal bands; NCS, nerve conduction studies; SSEPs, somatosensory evoked potentials; SSEPs-R, right-sided posterior tibial nerve SSEP; SSEPs-L, left-sided posterior tibial nerve SSEP; EEG, electroencephalography; MRI, magnetic resonance imaging; FM, foramen magnum. *9HPT time represents the mean of two trials for each hand. **25FTW time represents the mean of two trials. ***SDMT was administered in its oral form. ****BVMT-R total recall was calculated as the sum of three learning trials. *****CVLT-II total recall was calculated as the sum of five learning trials. ******Ophthalmological assessment comprised visual acuity, fundus examination, optical coherence tomography, and measurement of retinal nerve fiber layer/total nerve fiber layer thickness. *******Electrocardiography, echocardiography with strain analysis.

Given her family history, both her mother and maternal grandmother developed progressive gait and speech disturbances, cognitive impairment, and difficulties with memory and swallowing, and died at the ages 52 and 54, respectively. The exact causes of death of the patient’s mother and maternal grandmother are unknown. No formal diagnosis was established, and no genetic testing was performed due to the lack of molecular diagnostic availability at that time. The patient has no siblings and has two asymptomatic adolescent daughters, for whom predictive genetic testing has been deferred in accordance with current recommendations for late-onset hereditary transthyretin amyloidosis.

Abdominal fat pad aspiration and brain biopsy were performed; no amyloid deposits were detected in the fat pad specimen. Neuropathological examination of brain tissue obtained by neuro-endoscopic biopsy of the septum pellucidum revealed amorphous eosinophilic deposits. Congo red staining was positive, with characteristic apple-green birefringence under polarized light. Immuno-histochemical analysis demonstrated strong transthyretin positivity (TTR+++), while staining for serum amyloid A (AA), kappa, and lambda light chains was negative, confirming ATTR amyloid deposition. Nerve conduction studies were normal. Cardiac evaluation, including echocardiography and electrocardiography, was unremarkable. Electroencephalography showed no abnormalities. Based on these findings, a confirmed diagnosis of hereditary ATTR with leptomeningeal involvement was established.

Treatment with tolcapone 100 mg three times daily was initiated following diagnosis at the age of 43. The medication was well tolerated, with no abnormalities detected in laboratory tests. At the one-year follow-up, neurological status, cognitive function—particularly verbal learning—and certain functional abilities (mainly walking tests) had deteriorated. CSF analysis revealed a significant increase in protein levels, and follow-up spinal MRI demonstrated enlargement of the pre-existing myelopathic lesion with contrast enhancement (Table 1). In response, the tolcapone dose was increased to 200 mg three times daily. Liver function tests (ALT, AST, ALP, and total bilirubin) were assessed at baseline, monthly during the first 6 months of treatment, and every 3 months thereafter to monitor hepatic safety. No clinically significant elevations in ALT, AST, ALP, or bilirubin were observed, and no treatment discontinuations due to hepatic adverse events occurred.

Over the subsequent 4 years, the patient experienced progressive neurological decline, with further impairment of upper limb motor function, ambulation, and cognition—particularly visuospatial and verbal memory. CSF analysis revealed a substantial further increase in protein concentration, and SSEPs showed progressive prolongation of central conduction time on the left. Follow-up spinal MRI demonstrated ongoing progression of the intramedullary lesion, while cranial MRI remained largely unchanged, except for increased leptomeningeal enhancement and mildly more pronounced ependymal enhancement (Table 1, Figure 1). At the most recent follow-up, the patient was ambulatory for several 100 meters using two walking sticks. A wheelchair was required for longer-distance mobility.

## 4 Discussion

Leptomeningeal involvement represents a rare and severe manifestation of ATTRv. The *TTR* variant p.Ala45Thr is one of the few pathogenic *TTR* variants associated with CNS impairment (18–20). These TTR variants are highly destabilizing and can be secreted from the choroid plexus, manifesting in leptomeningeal amyloidosis (18). Here, we present five-year clinical, imaging, electrophysiological, and CSF-based insights in a patient with leptomeningeal ATTR treated with tolcapone, thereby expanding the phenotypic spectrum of the p.Ala45Thr variant and highlighting CNS vulnerability associated with this genotype.

Alternative disease-modifying therapies were also considered in the therapeutic context of ATTRv. Tafamidis, a clinically approved transthyretin stabilizer, represents a standard treatment for systemic ATTRv; however, its penetration into the central nervous system appears limited, and its efficacy in leptomeningeal disease remains uncertain. RNA-targeting therapies, such as patisiran and vutrisiran, effectively reduce hepatic TTR production but have limited or no meaningful blood–brain barrier penetration and are therefore unlikely to significantly affect leptomeningeal TTR production (21, 22). In this context, a CNS-penetrant TTR stabilizer such as tolcapone was considered a rational therapeutic option.

Tolcapone, a catechol-O-methyltransferase inhibitor recently identified as a potent transthyretin tetramer stabilizer (9–11), has demonstrated strong TTR-stabilizing properties *in vitro* and favorable CNS penetration (10, 11, 16). Tolcapone was initiated at 100 mg three times daily, based on the approved dosing regimen used in Parkinson’s disease, as no disease-specific dosing recommendations are currently available for ATTRv amyloidosis. After 1 year, the dose was increased to 200 mg three times daily because of ongoing disease progression and the absence of treatment-related adverse effects.

Although tolcapone has shown promise as a CNS-penetrant TTR stabilizer, clinical data on its long-term neurological efficacy in ATTRv remain limited. In the present case, the treatment was well tolerated, with no clinically significant laboratory abnormalities observed during follow-up. Nevertheless, the patient exhibited progressive neurological and cognitive deterioration, including worsening ambulation, impaired fine motor function, rising CSF protein levels, enlargement of the cervical spinal cord lesion on MRI, and prolonged central conduction times on SSEPs, indicating ongoing CNS disease progression despite treatment.

These findings provide valuable real-world, longitudinal evidence on treatment response and underscore the complexity of CNS ATTRv management. Multimodal follow-up—including MRI, CSF analysis, and electrophysiological assessments—enabled detailed evaluation of disease progression and therapeutic effects, highlighting the value of integrated monitoring in this rare population. Notably, progression observed during maximal tolcapone therapy may suggest that advanced CNS involvement could limit the potential impact of TTR-stabilizing strategies; however, this observation is based on a single case and does not allow conclusions regarding treatment efficacy or disease modification.

This report has limitations inherent to single-patient studies, including limited generalizability and the inability to establish causal relationships between treatment and disease course. At the time of treatment initiation, biomarker-based monitoring of CNS ATTRv was not part of routine clinical practice, and therefore no systematic assessment of transthyretin stabilization or neurodegeneration biomarkers was performed. In particular, while clinical and radiological deterioration was observed during follow-up, it remains unclear to what extent this reflects the natural course of leptomeningeal ATTRv versus a potential treatment-modified trajectory. Nevertheless, it represents the first longitudinal case providing detailed insights into CNS involvement, structural and biochemical changes, and functional outcomes in this genetic context. Despite these limitations, this clinical report offers valuable data to inform future studies and potential intervention strategies in this rare disease.

## 5 Conclusion

This case highlights progressive CNS involvement in TTR p.Ala45Thr-associated ATTRv despite CNS-penetrant TTR stabilization. The findings suggest ongoing disease activity despite treatment; however, no conclusions regarding treatment efficacy can be drawn from this single-case observation. These findings emphasize the need for systematic CNS surveillance and further evaluation of therapeutic strategies in leptomeningeal ATTRv.

## Data Availability

The datasets presented in this article are not readily available because of ethical and privacy restrictions. Requests to access the datasets should be directed to the corresponding author.
